# Evoked potentials and behavioral performance during different states of brain arousal

**DOI:** 10.1186/s12868-017-0340-9

**Published:** 2017-01-25

**Authors:** Jue Huang, Tilman Hensch, Christine Ulke, Christian Sander, Janek Spada, Philippe Jawinski, Ulrich Hegerl

**Affiliations:** 1 Department of Psychiatry and Psychotherapy, University of Leipzig, Semmelweisstrasse 10, 04103 Leipzig, Germany; 2 Depression Research Centre, German Depression Foundation, Leipzig, Germany

**Keywords:** VIGALL, Brain arousal, EEG-vigilance stage, Evoked potential, Oddball paradigm, Behavioral performance

## Abstract

**Background:**

Previous studies compared evoked potentials (EPs) between several sleep stages but only one uniform wake state. However, using electroencephalography (EEG), several arousal states can be distinguished before sleep onset. Recently, the Vigilance Algorithm Leipzig (VIGALL 2.0) has been developed, which automatically attributes one out of seven EEG-vigilance stages to each 1-s EEG segment, ranging from stage 0 (associated with cognitively active wakefulness), to stages A1, A2 and A3 (associated with relaxed wakefulness), to stages B1 and B2/3 (associated with drowsiness) up to stage C (indicating sleep onset). Applying VIGALL, we specified the effects of these finely differentiated EEG-vigilance stages (indicating arousal states) on EPs (P1, N1, P2, N300, MMN and P3) and behavioral performance. Subjects underwent an ignored and attended condition of a 2-h eyes-closed oddball-task. Final analysis included 43 subjects in the ignored and 51 subjects in the attended condition. First, the effect of brain arousal states on EPs and performance parameters were analyzed between EEG-vigilance stages A (i.e. A1, A2 and A3 combined), B1 and B2/3&C (i.e. B2/3 and C combined). Then, in a second step, the effects of the finely differentiated EEG-vigilance stages were further specified.

**Results:**

Comparing stages A versus B1 versus B2/3&C, a significant effect of EEG-vigilance stages on all behavioral parameters and all EPs, with exception of MMN and P3, was found. By applying VIGALL, a more detailed view of arousal effects on EP and performance was possible, such as the finding that the P2 showed no further significant increase in stages deeper than B1. Stage 0 did not differ from any of the A-stages. Within more fine-graded stages, such as the A-substages, EPs and performance only partially differed. However, these analyses were partly based on small sample sizes and future studies should take effort to get enough epochs of rare stages (such as A3 and C).

**Conclusions:**

A clear impact of arousal on EPs and behavioral performance was obtained, which emphasize the necessity to consider arousal effects when interpreting EPs.

**Electronic supplementary material:**

The online version of this article (doi:10.1186/s12868-017-0340-9) contains supplementary material, which is available to authorized users.

## Background

Brain arousal fundamentally impacts behavior and brain function, including evoked potentials (EPs), and is closely related to the sensitivity to external and internal stimuli [1]. However, the relation between brain arousal and sensory processing in the central nervous system is not fully understood, which is at least partly attributed to the lack of a valid and reliable tool to assess different brain arousal states at the appropriate scale.

Electroencephalography (EEG) is the gold standard to assess brain arousal. The most prominent classification of brain arousal by Rechtschaffen and Kales [2] distinguishes between relaxed wakefulness (stage W), non-rapid eye movement (NREM) sleep (stage I–IV) and rapid eye movement (REM) sleep. Previous studies applying the classification by Rechtschaffen and Kales demonstrated clear differences between wakefulness (i.e. stage W) and sleep (i.e. stage I–IV) concerning the amplitude of auditory EPs. From wakefulness to sleep stage II, an amplitude decrease was reported for N1 [3–6], mismatch negativity [MMN; [5, 7, 8] ], and P3 [3, 9–12], while the amplitude of P2 [3, 4, 13–15] and N300 [10, 12, 16] increased.

However, some contradictory results have also been reported. For example, Nittono et al. [17] failed to find any significant changes of the MMN amplitude across different arousal states. Similarly, discrepant results about the effect of brain arousal on the P1 amplitude were reported [18–20].

Several researchers suggested that sleep stage I can be divided into distinct substages [21–26]. For instance, Hori and colleagues classified nine stages with considerable stability [24], with the first two stages corresponding to stage W according to Rechtschaffen and Kales, stages 3–8 corresponding to sleep stage 1, and stage 9 corresponding to sleep stage 2 [24–27]. Significant differences in EPs between such substages have been reported [17, 21–23] and point to the need for subdividing the waking state before sleep onset.

Recently, the Vigilance Algorithm Leipzig (VIGALL) has been developed to objectively determine different brain arousal states during resting EEG recordings before sleep onset. The VIGALL has already been applied in studies with patients where the arousal regulation during resting EEG recordings might be a promising biomarker for differential diagnosis and treatment prediction [28–36]. VIGALL is an EEG- and electrooculography-based software, which objectively classifies brain arousal states by attributing one of seven EEG-vigilance stages to each 1 s (as default) EEG-segment [29, 37–39]. VIGALL takes into account the cortical distribution of EEG activity using source localization approaches [40, 41]. VIGALL is based on earlier EEG studies investigating the transition period between wakefulness and sleep [42, 43], which have been advanced in recent research [44–54]. As outlined in Table 1, VIGALL 2.0 (http://research.uni-leipzig.de/vigall/), differentiates the EEG-vigilance stage 0 (associated with cognitively active wakefulness), A1, A2, A3 (associated with relaxed wakefulness), B1, B2/3 (reflecting drowsiness) and C (indicating sleep onset).

**Table 1:** Assessment of brain arousal states by applying VIGALL 2.0

VIGALL stages	Corresponding behavioral state	EEG-characteristics
0	Cognitively active wakefulness	Low amplitude, desynchronized non-alpha EEG without horizontal SEM
A1		Occipital dominant alpha activity
A2	Relaxed wakefulness	Starting shifts of alpha to central and frontal cortical areas
A3		Continued frontalization of alpha
B1	Drowsiness	Low amplitude, desynchronized EEG with horizontal SEM
B2/3		Dominant delta- and theta-power
C	Sleep onset	Occurrence of K-complex and sleep spindles

*SEM* slow eye movements

To our knowledge, no studies have examined the effects of alterations in brain arousal on EPs by applying such a fine-graded classification system distinguishing different states of arousal on a second-by-second basis before sleep onset. To this end, we set out to specify the effects of alterations in brain arousal using VIGALL 2.0. With decreasing arousal (indicated by decreasing EEG-vigilance stages) we hypothesize an increase of P1, P2 and N300 amplitudes and a decrease of N1, MMN and P3.

## Methods

### Subjects

Healthy volunteers were recruited via local and online advertisements. Each subject gave written informed consent and was paid 20 €, or given course credits (psychology students) for participation. The study was approved by the local ethics committee of the University of Leipzig (075-13-11032013). All subjects were asked to participate in two EEG recordings (one ignored and one attended oddball condition) with an interval of seven days between sessions. The sequence of the ignored and attended conditions was balanced between subjects. However, not all subjects participated in the second session due to lack of compliance or availability, leaving 49 subjects in the ignored and 54 subjects in the attended condition.

None of subjects reported a history of sleep disorder or psychiatric or neurological diseases or current intake of psychotropic medication. Subjects exhibiting alpha variant or low voltage EEGs (n = 2 in the ignored condition); excessive movement artifacts (>50%; n = 1 in the ignored condition); insufficient arousal variability during the 2 h recording (n = 2 in the ignored and attended conditions, respectively) and unusual sleeping behavior (the eyes were partially open during sleep in one subject) were excluded. The final sample included 43 subjects in the ignored (26 female, age = 23.8 ± 3.8, aged from 18 to 33 years) and 51 subjects in the attended condition (31 female, age = 24.5 ± 4.4, aged from 18 to 34 years).

### Procedure

The 2-h EEG recordings began between 1 and 4 p.m. For each individual the time of assessment was the same in both sessions. EEGs were recorded within a light dimmed and sound attenuated booth with a maintained temperature below 25 degrees Celsius.

During the EEG, subjects lay comfortably on a lounger with closed eyes while tones of an oddball paradigm were presented. Subjects were instructed to relax and explicitly allowed to follow their own natural course of wakefulness decline. In the case of subjects falling asleep, they were woken up after 5 min and asked to answer a common question (e.g. today’s date). Subsequently, they were allowed to continue the task. This process was repeated until the end of the experiment in order to acquire enough data from all of the arousal states.

### Oddball paradigm

A standard (500 Hz) and a deviant (1000 Hz) tone were presented in a classic oddball pattern [55] with stimuli probability of 80% and 20% respectively. Each deviant stimulus was preceded by at least two standard stimuli. Each stimulus had a duration of 50 ms and an intensity of 70 dB SPL. Stimuli had a randomized inter-stimulus interval between 900 and 1400 ms. Subjects were instructed to ignore the tones under the ignored condition and, under the attended condition, to press a button with their dominant hand every time a deviant (target) tone was presented. Stimuli were presented binaurally via insert earphones (E-A-RTONE 3A, Aearo Company Auditory System, Indianapolis, IN, USA) using Presentation software (Presentation, Neurobehavioral Systems). The simultaneity of trigger and sound was confirmed following Neurobehavioral Systems’ guideline (https://www.neurobs.com/menu_presentation/menu_hardware/system_configuration).

### EEG-recording procedure and EEG-vigilance staging

The EEG was recorded with Ag/AgCl electrodes and QuickAmp amplifiers (24 bit; DC and 200 Hz low pass; Brain Products GmbH, Gilching, Germany) from 31 sites (Fp1, Fp2, F3, F4, F7, F8, Fz, FC1, FC2, FC5, FC6, C3, C4, T7, T8, Cz, FT9, FT10, CP5, CP6, TP9, TP10, P3, P4, P7, P8, Pz, O1, O2, PO9, PO10) according to the extended international 10–20 system using EasyCap (EASYCAP Brain Products GmbH, Gilching, Germany), and referenced against the common average. Impedance of each electrode was kept below 10 kΩ and sampling rate was 1000 Hz. A bipolar electrode placed lateral of the left and right eye served to monitor horizontal eye movements. Another bipolar electrode was placed above and below the right eye to monitor vertical eye movements.

EEG data were analyzed using BrainVision Analyzer software (Brain Products GmbH, Gilching, Germany). First, the EEG raw data was pre-processed according to standard operating procedure (SOP; see section EEG preprocessing in Additional file 1 and VIGALL manual available at http://research.uni-leipzig.de/vigall/). After that, VIGALL 2.0 was used to classify the respective EEG-vigilance stage in the interval of 500 ms before each stimulus. Results of the EEG-vigilance analysis were exported as markers to be used in the EP analysis.

### EP analysis

The EEG raw data with imported vigilance markers were filtered offline with a bandpass filter of 0.5–30 Hz. The EEG was divided into 900 ms epochs (100 ms pre and 800 ms post stimulus) time locked to the onset of each auditory stimulus. Standard stimuli that immediately followed a deviant stimulus were discarded from analysis. Epochs were rejected if the EEG amplitude exceeded ±100 µV. Baseline correction was applied for the 100 ms pre stimulus interval. Subsequently grand averages for standard, deviant and difference waveforms were calculated separately for each EEG-vigilance stage (for subjects with at least 50 epochs). Peaks were detected by Vision Analyzer’s inbuilt peak detection module based on search windows derived from visual inspection of grand average waveforms. Then, for each component, the mean value of a given time window around the peak was exported for statistical analyses (for details see section EP parameterization in Additional file 1).

In the current study, analyses for P3 were not limited to detected target stimuli for two reasons: First, the number of subjects who had sufficient epochs for each EEG-vigilance stage and also showed responses to the target tone was too low (n = 8, even in the first analysis step comparing A vs. B1 vs. B2/3&C; see statistical analysis below). Secondly, in the current paradigm, a lack of response does not automatically imply that the subjects failed to detect a target stimulus, especially in a drowsy state. Subjects were allowed to relax and to fall asleep; thus they might have given up making overt responses in order to fall asleep, although they might have still detected the target. To assess the MMN in the ignored condition, the difference waveform was calculated by subtraction of EPs to standard stimuli from EPs to deviant stimuli. For MMN detection we took into account a time window between 100 and 150 ms post stimulus. Analyses for EPs were done at the respective gold-standard electrode positions, i.e. Fz for MMN, Pz for P3, and Cz for all other EPs.

### Behavioral data analysis

The behavioral data analysis was processed in Matlab (Mathworks, Natick, MA). We defined a hit as correct response (key press) between 50 and 900 ms after target stimulus. Hit rate (HR) was defined as the percentage of hits in relation to all target stimuli. Reaction time (RT) was defined as the average time interval between target stimulus and correct response. Omission rate (OR) was the percentage of missing responses to targets in relation to all target stimuli. False alarm rate (FAR) referred to the percentage of key presses to non-target stimuli in relation to the total number of non-target stimuli.

### Statistical analysis

In order to get reliable EPs, a minimum criterion of 50 epochs for each EEG-vigilance stage was set. Subjects with insufficient number of epochs were excluded from the analyses of the respective stage. To examine the effect of brain arousal states on EPs and behavioral performance, a repeated measures ANOVA and paired sample *t* tests were run. All statistical analyses were processed using IBM SPSS Statistics version 20 (IBM, Armonk, NY, USA).

The number of subjects reaching the 50 epoch criterion in the respective EEG-vigilance stages is shown in Additional file 2: Table S1. Some stages, such as A1, were highly frequent, whereas others, especially A3 and C, rarely occurred. The rare occurrence of some EEG-vigilance stages prohibited an analysis of the effect of all EEG-vigilance stages at once within one repeated measures ANOVA with seven factor levels. Therefore, all analyses were done with separate stepwise within-subjects analyses in order to get sufficiently powered comparisons, each based on the same subjects. This within-subjects approach was done to avoid a systematic bias, because the speed with which one enters into low arousal states can be considered a personality trait [29, 56, 57]. Those who rapidly enter into low arousal states will be overrepresented in groups of subjects who, for example, fulfill the criterion of more than 50 epochs of the low arousal stage “C”. However, these subjects with an unstable arousal regulation have been characterized by personality traits, which are also associated with EP amplitude peculiarities [57–59]. As a consequence, differences in EPs found between different arousal states could simply be due to preexisting trait differences. Additionally, the EEG-vigilance stage groups might also differ concerning such variables as sex and age [60], which are also associated with EP amplitudes [61–63]. To avoid these systematic biases, the following steps of analyses were run, in each case based on the same subjects:

1. First, the effect of brain arousal states on EPs and behavioral parameters was analyzed in a repeated measures ANOVA with factor *EEG*-*Vigilance stages*, comprising stage A (i.e. A1, A2 and A3 combined), B1 and B2/3&C (i.e. B2/3 and C combined). Greenhouse–Geisser correction of degrees of freedom (df) was applied if result of sphericity test was significant. Where the main effect was significant, a post hoc test for multiple comparisons was conducted with adjustments for significance level using the Bonferroni method.
2. Secondly, differences between the following EEG-vigilance stages were analyzed by paired sample *t* tests (for the numbers of included subjects for each comparison see Additional file 2: Table S2): As stage 0 had been excluded in the first step, the differences of 0 versus A1, 0 versus A2 and 0 versus A3 were compared. The differences between A-substages were further specified by three paired sample *t* tests. As B2/3 and C had also been pooled together in the first step, we finally investigated the differences between B1, B2/3 and C by three paired sample t-tests.

## Results

In the following, results for the EPs P1, N1, P2 and N300 are reported for standard stimuli, which is the gold standard for these components. In addition, we also calculated the EPs from deviant stimuli for these components, and results were similar and can be found in Additional file 3: Figure S1 (for the ignored condition) and Figure S2 (for the attended condition). The results for P3, MMN and performance data will thereafter be reported together because they all are derived from deviant stimuli.

### EPs to standard stimuli

#### EPs between stages A, B1 and B2/3&C

**Fig. 1:**
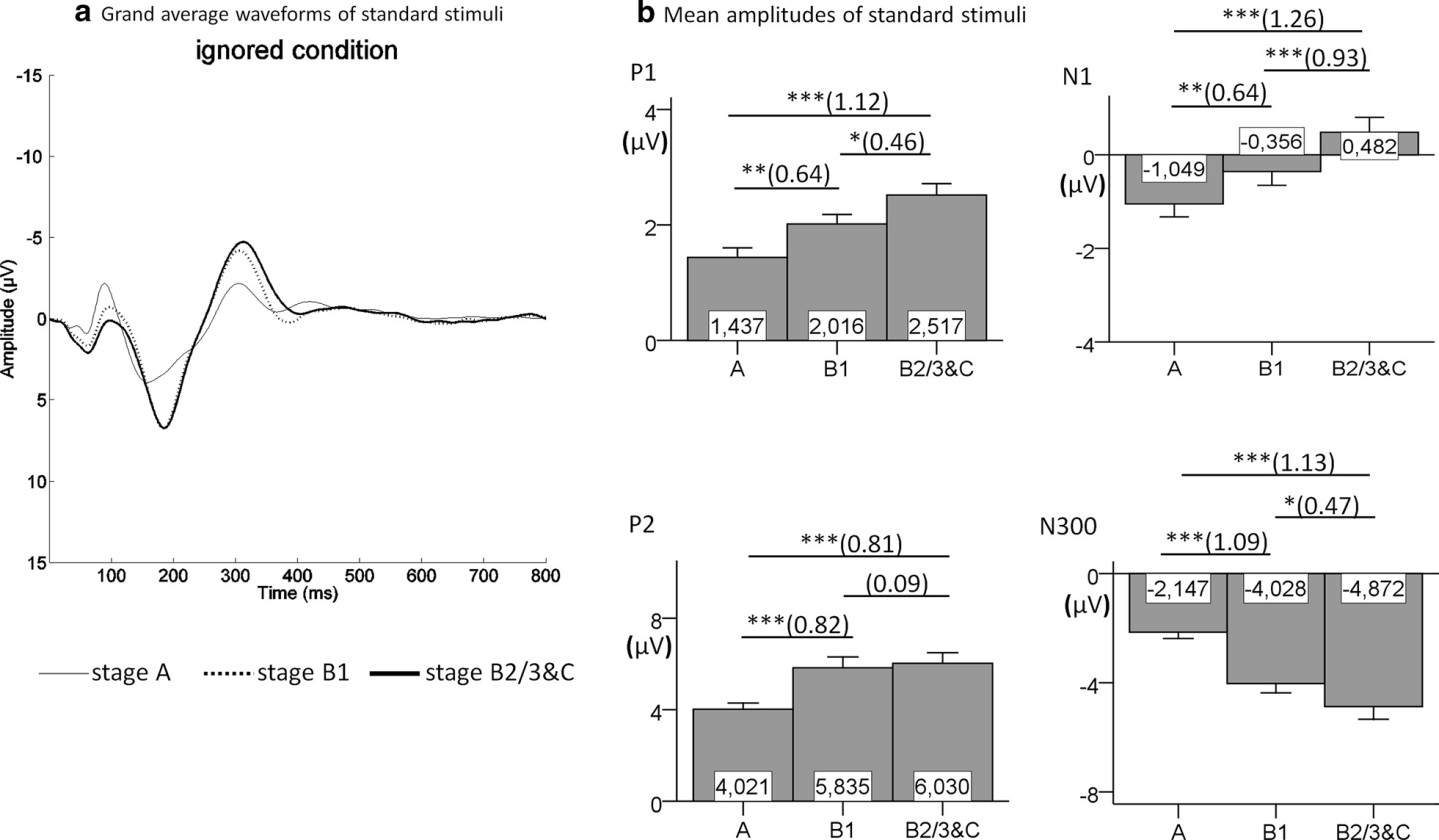
Grand average waveforms (**a**) and mean amplitudes (**b**) for standard components in the ignored condition. The standard *P1*, *N1*, *P2* and N300 are presented at Cz electrode in EEG-vigilance stages *A*, *B1* and *B2/3&C* (N = 38). The significant results of multiple comparisons are marked with *asterisk* (*p < .05; **p < .01; ***p < .001; each *p* value is Bonferroni corrected). The corresponding effect sizes for Cohen’s *dz* are represented in *parentheses*

**Fig. 2:**
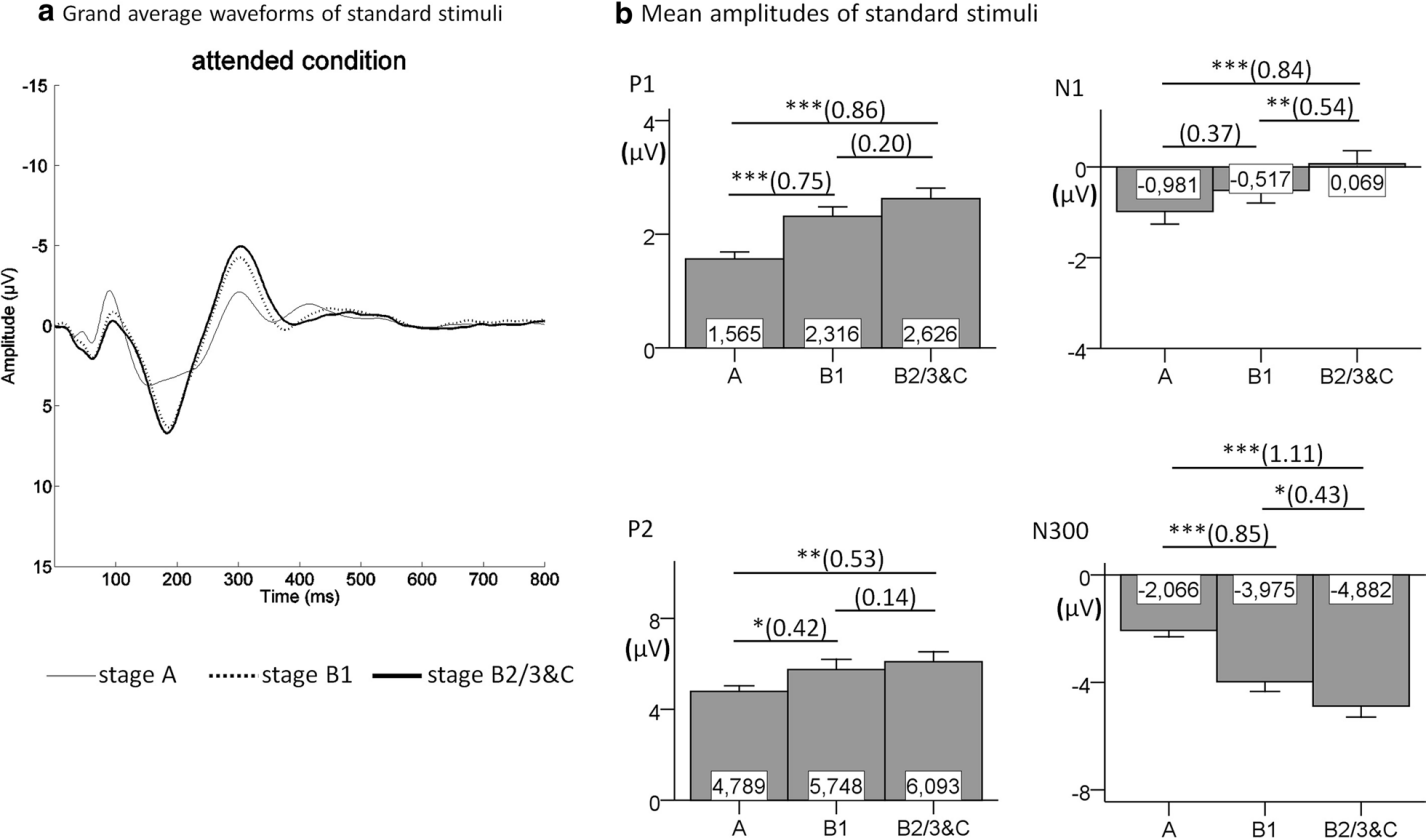
Grand average waveforms (**a**) and mean amplitudes (**b**) for standard components in the attended condition. The standard *P1*, *N1*, *P2* and N300 are presented at Cz electrode in EEG-vigilance stages *A*, *B1* and *B2/3&C* (N = 44). The significant results of multiple comparisons are marked with *asterisk* (*p < .05; **p < .01; ***p < .001; each *p* value is Bonferroni corrected). The corresponding effect sizes for Cohen’s *dz* are represented in *parentheses*

The grand average waveforms and mean amplitudes of P1, N1, P2 and N300, which were elicited by standard stimuli, as well as the corresponding results of multiple comparisons between EEG-vigilance stages are illustrated in Fig. 1 for the ignored condition and in Fig. 2 for the attended condition. The main effects of EEG-vigilance stages on all EPs reached statistical significance: In the ignored condition, there was a main effect of EEG-vigilance stages on the amplitudes of P1 [*F*_(2,74)_ = 22.532, *p* < .001, $\eta_{p}^{2}$ = 0.378], N1 [*F*_(2,74)_ = 38.548, *p* < .001, $\eta_{p}^{2}$ = 0.510], P2 [*F*_(2,74)_ = 17.707, *p* < .001, $\eta_{p}^{2}$ = 0.324] and N300 [*F*_(1.668,61.725)_ = 37.029, *p* < .001, $\eta_{p}^{2}$ = 0.500]; in the attended condition, the amplitudes of P1 [*F*_(1.594,68.554)_ = 15.972, *p* < .001, $\eta_{p}^{2}$ = 0.271], N1 [*F*_(2,86)_ = 17.038, *p* < .001, $\eta_{p}^{2}$ = 0.284], P2 [*F*_(2,86)_ = 7.043, *p* < .01, $\eta_{p}^{2}$ = 0.141] and N300 [*F*_(2,86)_ = 34.419, *p* < .001, $\eta_{p}^{2}$ = 0.445] also differed significantly between the EEG-vigilance stages. The results for multiple comparisons were all in the expected direction and were significant with the exception of the difference between B1 and B2/3&C for P2 (both conditions) and P1 (attended condition). Additionally, the difference between A and B1 for the attended condition for N1 failed to reach significance level (*p* = .052, *dz* = 0.37).

#### EPs between stages 0 versus A1, 0 versus A2 and 0 versus A3

Except for P2 (all *p* < .05) in the attended condition, no significant differences between the stages were found (see Additional file 4: Table S3).

#### EPs between A-substages

Only some differences between A-substages were significant (see Additional file 4: Table S4), mostly for P1 and P2, which both significantly differed between A1 and A3 in both ignored and attended conditions (all *p* < .05) with effect sizes *dz* between 0.48 and 0.57. In the attended condition, P2 significantly differed between A1 and A2 (*p* < .05, *dz* = 0.33) and P1 between A2 and A3 (*p* < .01, *dz* = 0.63).

#### EPs between stages B1, B2/3 and C

In both conditions, N1 and N300 significantly differed in the expected direction between B1 and B2/3 (all *p* < .05) and also between B1 and C (all *p* < .05) with effect sizes *dz* between 0.35 and 1.07. The same was the case for P1 (all *p* < .05, *dz* between 0.36 and 0.79), with exception of B1 versus B2/3 in the attended condition, which did not show significant differences (*dz* = 0.13). P2 did not significantly differ between B1 and B2/3 or between B1 and C (*dz* between 0.08 and 0.17). Concerning the comparison of B2/3 with C (*dz* between 0.03 and 0.44), no component reached significance. Detailed results for comparisons are shown in Additional file 4: Table S5.

### EPs and behavioral performance to deviant stimuli

#### EPs and behavioral performance between stages A, B1 and B2/3&C

**Fig. 3:**
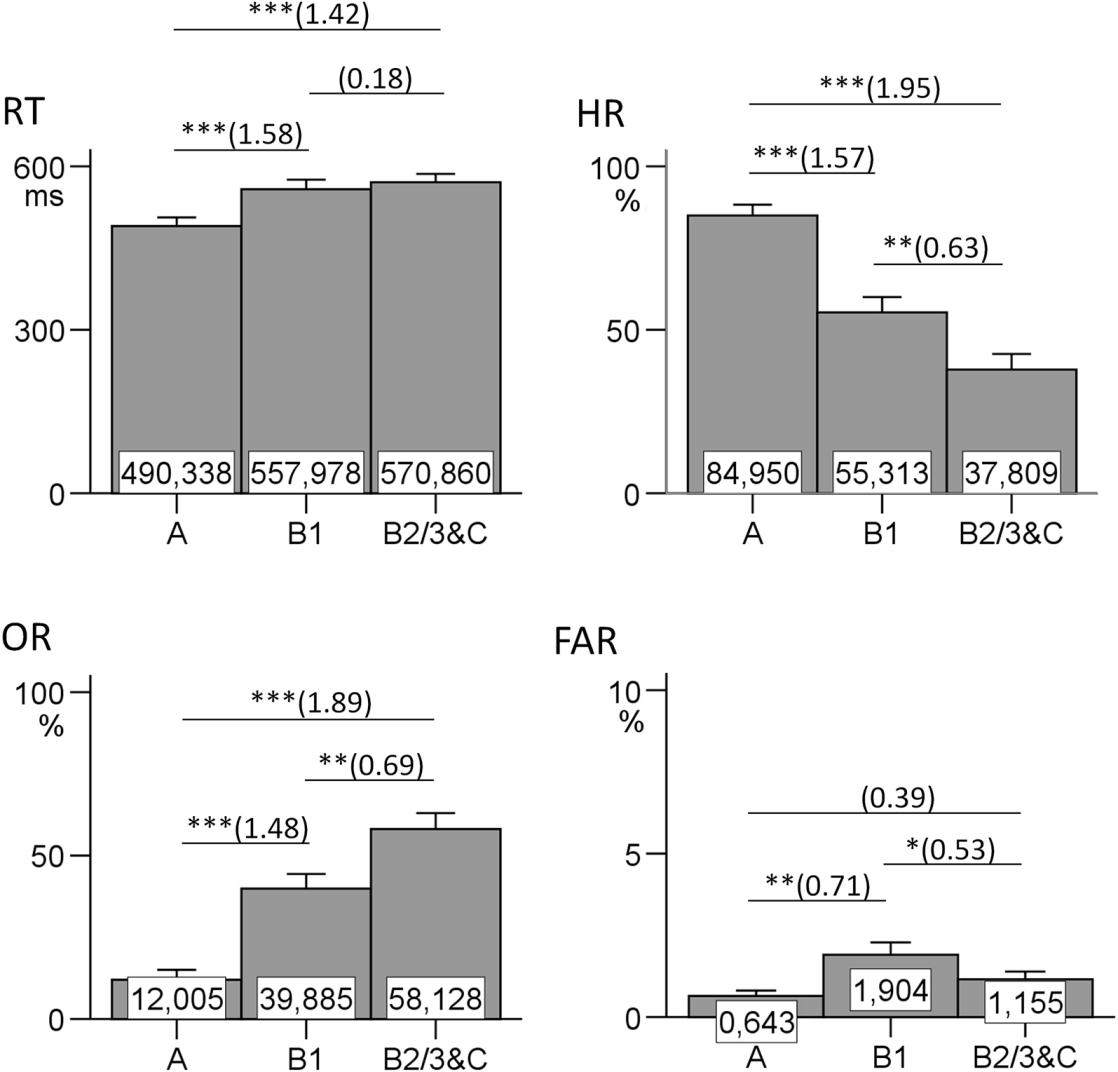
Behavioral performance concerning target stimuli in the attended condition. The averaged reaction time (*RT*), hit rate (*HR*), omission rate (*OR*) and false alarm rate (*FAR*) are shown in EEG-vigilance stages *A*, *B1* and *B2/3&C* (N = 29). The significant results of multiple comparisons are signed with *asterisk* (*p < .05; **p < .01; ***p < .001; each *p* value is Bonferroni corrected). The corresponding effect sizes for Cohen’s *dz* are represented in *parentheses*

There was no effect of EEG-vigilance stages on the amplitudes of MMN [*F*_(2,60)_ = 0.126, *p* = .882, $\eta_{p}^{2}$ = 0.004] in the ignored condition or on P3 in the attended condition [*F*_(2,56)_ = 1.416, *p* = .251, $\eta_{p}^{2}$ = 0.048]. However, the performance parameters differed significantly among EEG-vigilance stages (see Fig. 3): RT [*F*_(1.537,43.031)_ = 32.197, *p* < .001, $\eta_{p}^{2}$ = 0.535], HR [*F*_(2,56)_ = 57.579, *p* < .001, $\eta_{p}^{2}$ = 0.637], OR [*F*_(2,56)_ = 56.933, *p* < .001, $\eta_{p}^{2}$ = 0.670] and FAR [*F*_(2,56)_ = 10.279, *p* < .001, $\eta_{p}^{2}$ = 0.269]. The results of multiple comparisons between stages are illustrated in Fig. 3. As no effect of EEG-vigilance stages on the MMN and P3 was found, paired sample *t* tests were only done for behavioral parameters.

#### Behavioral performance between stages 0 versus A1, 0 versus A2 and 0 versus A3

No behavioral parameter showed a significant difference between stages, nor did we observe any consistent increase or decrease from high to low stages. However, it should be noted that analyses for the comparison 0 versus A3 were not done due to the insufficient sample size (N = 3). The detailed results are presented in Additional file 4: Table S3.

#### Behavioral performance between A-substages

Although some behavioral parameters showed a trend of performance decline with declining of A-substages, only the HR differed significantly between A1 and A2 (*p* < .05; *dz* = 0.37) and between A1 and A3 (*p* < .05, *dz* = 0.79). The detailed results are presented in Additional file 4: Table S4.

#### Behavioral performance between stages B1, B2/3 and C

Compared with B1, an impaired performance was found in B2/3 for HR (*p* < .01, *dz* = 0.61), OR (*p* < .01, *dz* = 0.68) and FAR (*p* < .05, *dz* = 0.44), whereas RT did not significantly differ (*dz* = 0.16). Comparisons with stage C were not possible, since the number of subjects was insufficient (N = 7). The detailed results are presented in Additional file 4: Table S5.

## Discussion

### Analyses of EEG-vigilance stages A, B1 and B2/3&C

The present study clearly showed an effect of brain arousal on sensory processing as reflected by EPs. The amplitudes of P1, N1, P2 and N300 were significantly associated with EEG-vigilance stages when compared in stages A, B1 and B2/3&C. As expected, a continuous amplitude increase of P1 and N300 during declining EEG-vigilance stages was found, with the only exception in the attended condition, where the comparison of B1 versus B2/3&C for P1 failed to reach significance. Replicating previous studies [64, 65], the N300 increased its amplitude as brain arousal decreased. The P2, as expected, also increased with declining EEG-vigilance stages. However, the P2 was only sensitive towards higher EEG-vigilance stages, and showed no further increase after reaching stage B1. Also as expected, the N1 became smaller with decreasing EEG-vigilance stages, with the only exception in the attended condition, where the comparison of A versus B1 only showed a tendency in expected direction (*p* = .052 after Bonfferoni correction). The effect size *dz* for this comparison was 0.37, thus one might hypothesize that a larger sample size or longer recording time might lead to this comparison becoming significant. The performance data were all in expected direction, and main effects, as well as multiple comparisons, were significant—except that FAR was slightly lower in B2/3&C than in B1, which might reflect a less impulsive response style in very low arousal states. Additionally, RT between B1 and B2/3&C did not significantly differ. This might be due to the fact that subjects were allowed to relax and fall asleep. Therefore, some subjects might have started to deliberately react slowly in early drowsy states, obscuring differences between B1 and deeper arousal states.

In this study, no effect of EEG-vigilance stages on the MMN was found. This is consistent with results found by Nittono et al. [17] and Sabri et al. [66], which, as did the current study, used a frequency oddball paradigm. However, Jacobsen and Schröger [67] suggested that the frequency oddball paradigm may not elicit a pure MMN, particularly when the extent of deviance is large. Therefore, a deviance-related negativity [68] that is composed of both the MMN and N1 might have occurred in this study. Our deviance-related negativity showed some characteristics of a true MMN (largest deflection at Fz, within MMN time window, polarity inverse at mastoids), however, due to spatial and temporal overlapping, the separation of the MMN from N1 is very difficult. To give a definitive answer to the question of whether the MMN is sensitive to the brain arousal states, a control condition suggested by Jacobsen and Schröger [69], where all stimuli are presented randomly with equal probability, is suggested for further studies.

The MMN in this study was calculated by subtracting EPs to standard stimuli from EPs to deviant stimuli. An effect of brain arousal on the MMN will therefore only be visible when brain arousal has impacts on either the deviant or standard EPs. Additionally, by calculating the difference, reliability of the MMN might be impaired [70], constraining the possibility for an association with EEG-vigilance stages. In general, it is a limitation of the current study that, although the EPs show acceptable reliabilities under standard conditions [61, 71], and also the VIGALL has proved its reliability during 20-min recording [37], nothing is known about reliabilities of EPs in the current paradigm (2 h recording in drowsy state with closed eyes).

As outlined in previous reviews [72, 73], P3 is suggested to be related to arousal fluctuations. However, in this study no effect of EEG-vigilance stages on P3 was observed. One reason for this might be that, in contrast to previous studies [9, 10], no differentiation of P3 to detected target stimuli from that to non-detected was done. As mentioned before, too few subjects remained when only detected stimuli went into analyses. Additionally, in the current paradigm, where subjects were allowed to relax and fall asleep, no reaction would have not automatically implied an absence of detection. Another reason, why other studies might have successfully found an arousal effect on P3 is recording time, which lasted from 3 h to overnight [3, 9, 10, 22]. Thus, the sample size and/or the recording time might have to be larger than in the current study in order to detect small differences of the P3 between EEG-vigilance stages.

### Analyses of EEG-vigilance stage 0

Comparisons of EEG-vigilance stages done by paired t-tests were partly hampered by small sample sizes. Future studies should take effort to get enough epochs of rare stages, e.g. by longer EEGs, such as an overnight EEG. Nonetheless, some preliminary conclusions concerning EPs in different EEG-vigilance stages can be drawn. In this study, there was no clear evidence for EPs or behavioral performance differing between stages 0 and A1, A2, or A3, respectively. Stage 0 has only recently been added to the VIGALL in order to differentiate desynchronized non-alpha EEG (stage 0) indicating active wakefulness from a similar low-amplitude EEG pattern (stage B1) indicating drowsiness. Thus, for the sake of completeness, we therefore also calculated whether the EPs or performance parameters differ significantly between stages 0 and B1. As expected, most differences were significant (see Additional file 4: Table S6).

### Analyses of EEG-vigilance A-substages

Concerning the comparisons between A-substages, only some EPs and performance parameters were significant. Given the scattered significances, the current study does not clarify whether EPs or performance really differ between subtle arousal differences, reflected by A-substages. Considering the small subject number and other inherent limitations in analyses discussed below, further studies are needed to answer this question.

### Analyses of EEG-vigilance stages B1, B2/3 and C

Analyses of stages B1, B2/3 and C confirmed the finding of the main analyses based on combined stages that the P2 brain arousal association is limited to high arousal levels. The P2 amplitude did not differ between any of the low EEG-vigilance stages (B1, B2/3 and C). All other components differed between B1 and B2/3 as well as between B1 and C, but not between B2/3 and C. This unexpected finding of no difference between B2/3 and C might be explained (already taking into consideration the small sample size of this comparison) by the way stage C was classified: When signs of sleep (K-complexes or sleep spindles) were present in an EP segment, the 30 following segments were classified as belonging to stage C, unless criteria for an A-substage were fulfilled within these 30 segments (in this case, stage C classification was ended with the preceding segment and the A-substage was classified). Thus, within a sequence of stage C segments, B-stages can possibly be embedded, which might blur the difference between B2/3 and C.

Concerning the behavioral performance, only the comparison of B1 versus B2/3 had a sufficient subject number and revealed significant differences for HR, OR and FAR, however, not for RT. As discussed above, the lack of RT differences might be due to the instruction that subjects were allowed to relax and fall asleep, leading to less clear RT-differences between B1 and deeper arousal states.

### Limitations

A limitation inherent in the current experimental design is that EEG-vigilance stages are assessed in an oddball paradigm, which differs from the SOP ideally used to assess arousal regulation. Following the VIGALL SOP (http://research.uni-leipzig.de/vigall/) the resting EEG should be recorded during strict quietness without any task so that the subjects can follow their natural course of arousal decline. In contrast, during the current oddball paradigm, tones were presented. Additionally, in the attended condition, subjects were asked to press a button in response to the target tone as long as they were awake. Thus, although subjects were allowed to fall asleep in the attended condition, they nonetheless executed a simple task. Cognitive tasks have been suggested to induce “cognitive” theta and/or alpha frequencies [74], which may then not indicate arousal and thus possibly affect validity of VIGALL classification under such circumstances. However, in the current study the associations of EEG-vigilance stages with EPs were on the whole comparable for the attended and ignored conditions, which suggest that the instruction might not have significantly affected results.

Similarly, the oddball paradigm had to be adapted to the current aim of the study. The recording was done with moderate tone intensity and closed eyes in order to follow the VIGALL SOPs of arousal assessment as far as possible. However, there is some evidence that recording with closed eyes does not compromise the standard oddball condition [75]. In addition, the typical duration was largely extended (compared to standard oddball paradigms) in order to get enough EEG-vigilance stage variability. These changes might have reduced EPs amplitudes and their reliabilities.

Finally, one might argue that the association between EPs/performance and EEG-vigilance stages might partly reflect habituation and exhaustion effects, because the longer the recording duration the more habituation, exhaustion and the more low arousal states might occur. However, there are several arguments that the EP and performance changes mainly result from arousal changes. Firstly, our study recording time was still moderate, compared with recording times of either 3 h or over the course of several nights that is the recording time of other studies [3, 9, 10, 22]. Secondly, subjects were awoken and activated in each case where sleep lasted more than 5 min. Therefore, during the 2 h EEG-vigilance stages were quite equally distributed (see Additional file 5: Table S7), which can be expected to avoid a confounding of EEG-vigilance stages with time-on-task effects. Finally, in order to further verify that arousal effects on EPs occur independently of habituation effects, we analyzed the effects of brain arousal on standard N1, while reducing time-on-task effects as outlined in the following. We chose the N1 because this component is very strongly affected by habituation [76]. We segmented our entire EEG segment into four 30-min-blocks (minute 1–30, 31–60, 61–90 and 91–120). We then computed the averaged amplitudes for standard N1 during stages A and B2/3&C within each time block (i.e. $\overline{N1}$_A,block1_, …; $\overline{N1}$_B2/3&C,block1_, …), respectively (at least 20 epochs in the respective stage and in each time block were required). To control the effect of time-on-task during the 2 h, the averages of averages (from block 1 to 4) during stages A and B2/3&C were then computed. In the ignored condition, $\overline{{{\text{N}}1}}$_A(block1-4)_ was still significantly larger than $\overline{{{\text{N}}1}}$_B2/3&C(block1-4)_ [$\overline{N1}$_A(block1-4)_ = −1.7 µV, $\overline{N1}$_B2/3&C(block1-4)_ = −0.6 µV, *t*_(24)_ = −3.940, *p* < .01]; and in the attended condition, similar difference has been obtained [$\overline{N1}$_A(block1-4)_ = −1.4 µV, $\overline{N1}$_B2/3&C(block1-4)_ = − 0.3 µV, *t*_(21)_ = −4.616, *p* < .001]. These results together with the equal distribution of arousal states across the 2 h, suggest that the decrement of EPs during lower EEG-vigilance stages is caused not only by time-on-task, it was also caused by the decline of EEG-vigilance stages.

## Conclusion

In conclusion, for the first time, the present study demonstrated the sensitivity of EP components and behavioral performance to EEG-vigilance stages A, B1 and B2/3&C, thereby contributing to the validation of VIGALL. The directions of the EP-arousal associations were as expected from previous studies, which applied less fine-graded arousal classifications. With decreasing arousal, a decrease of N1 and an increase of P1, P2 and N300 were found. By applying VIGALL, a more detailed view on these arousal associations was possible, such as the finding that P2 showed no further amplitude increase in stages deeper than B1. In the second step of analyses, no differences in EPs and performance could be shown for stage 0 compared with A-stages. However, the sensitivities of EPs and performance to the other single stages (A-substages, B1, B2/3 and C) have been partly confirmed. As limitation of the current study, those analyses comparing single stages were partly based on small sample sizes and future studies should take effort to get enough epochs of these stages, e.g. by an overnight EEG. Nonetheless, the main findings of a clear arousal dependency of EPs and performance clearly point to the necessity to control or consider arousal effects when interpreting EPs.

## Additional files

**Additional file 1.** EEG preprocessing and EP parameterization.

**Additional file 2.** Number of subjects in each EEG-vigilance stage and number of included subjects for EEG-vigilance stage comparisons.

**Additional file 3.** Grand average waveforms for deviant stimuli and results of multiple comparisons.

**Additional file 4.** Paired sample t-tests for comparisons of EPs and behavioral performance between EEG-vigilance (sub-)stages.

**Additional file 5.** Mean number of epochs in EEG-vigilance stages during 4 time blocks.

## Abbreviations

EP: evoked potential
EEG: electroencephalography
VIGALL: Vigilance Algorithm Leipzig
NREM: non-rapid eye movement
REM: rapid eye movement
MMN: mismatch negativity
SEM: slow eye movement
SOP: standard operating procedure
HR: hit rate
RT: reaction time
OR: omission rate
FAR: false alarm rate
df: degrees of freedom
