# Supplementary material for: Evoked potentials and behavioral performance during different states of brain arousal
Source: BMC Neurosci. 2017 Jan 25;18:21. doi: 10.1186/s12868-017-0340-9 (PMC5267455; doi:10.1186/s12868-017-0340-9)
Supplement: Supplementary file 1 — Additional file 1. EEG preprocessing and EP parameterization. [file 12868_2017_340_MOESM1_ESM.docx]

**EEG preprocessing**

EEG preprocessing was done applying Vision Analyzer (Brain Products GmbH, Gilching, Germany) with the following steps:

1) An offline bandpass filter between 0.5-70Hz (with notch-filter at 50Hz) was applied to the EEG and EOG data, with exception that there was no high-pass filter for EOG.

2) The 2 hours EEG data was then divided into equal 1-sec segments.

3) Very obvious muscle, swallow, eye movements and sweating artifacts were marked through rough visual artifact screening.

4) Thereafter, an independent components analysis was performed. The eye movements and continuous muscle artifacts were removed by extracting independent components that clearly contained only artifact-related information.

5) The EEG segments were again visually screened in-depth for the remaining artifacts that were not removed by rough artifact screening and ICA.

6) The classification of EEG-vigilance stage C via the VIGALL relies on the occurrence of graph elements indicating sleep onset (i.e. K-complex or sleep spindles), therefore all EEGs had visually been screened for such graph elements and the respective segments had been manually marked. This step can also be performed before step 4.

7) In order to increase processing speed, the sampling rate was changed to 100 Hz.

8) Afterwards, the already segmented epochs in step 2 were reversed because VIGALL requires a continuous EEG.

**EP parameterization**

The search window for each evoked potential was based on the latency in grand average. The detailed search windows are presented in Table below. Automatic peak detection module of Vision Analyzer (Brain Products GmbH, Gilching, Germany) was used for peak detection.

|  | ignored condition | | |  | attended condition | | |
| --- | --- | --- | --- | --- | --- | --- | --- |
| Evoked potential | Start (ms) | End (ms) | Polarity (+/-) |  | Start (ms) | End (ms) | Polarity (+/-) |
| P1 | 34 | 84 | + |  | 33 | 83 | + |
| N1 | 64 | 124 | - |  | 65 | 125 | - |
| P2 | 130 | 230 | + |  | 119 | 219 | + |
| N300 | 270 | 350 | - |  | 268 | 348 | - |
| P3 |  |  |  |  | 320 | 500 | + |

Thereafter, the mean amplitude values within given windows around the identified peaks were exported of each component for statistical analyses: The windows were: P1 (± 5 ms), N1 (± 25 ms), P2 (± 30 ms), N300 (± 25 ms) and P3 (± 20 ms)
