# Supplementary material for: Evoked potentials and behavioral performance during different states of brain arousal
Source: BMC Neurosci. 2017 Jan 25;18:21. doi: 10.1186/s12868-017-0340-9 (PMC5267455; doi:10.1186/s12868-017-0340-9)
Supplement: Supplementary file 2 — Additional file 2. Number of subjects in each EEG-vigilance stage and number of included subjects for EEG-vigilance stage comparisons. [file 12868_2017_340_MOESM2_ESM.docx]

Table S1. Number of subjects and epochs in the respective EEG-vigilance stage

| **EEG-vigilance**  **(sub)stages** | **ignored condition** | | | | | | |  | **attended condition** | | | | | | |
| --- | --- | --- | --- | --- | --- | --- | --- | --- | --- | --- | --- | --- | --- | --- | --- |
|  | **standard stimuli** | | |  | **deviant stimuli** | | |  | **standard stimuli** | | |  | **deviant stimuli** | | |
|  | **n** | **range** | **mean** |  | **n** | **range** | **mean** |  | **n** | **range** | **mean** |  | **n** | **range** | **mean** |
| 0 | 24 | 54-1058 | 327.1 (264.4) |  | 18 | 50-339 | 135.9 (83.2) |  | 31 | 51-1843 | 395.0 (416.0) |  | 22 | 51-638 | 182.1 (144.1) |
| A1 | 40 | 62-2599 | 1200.0 (631.5) |  | 39 | 61-872 | 408.6 (207.5) |  | 51 | 132-2884 | 1277.6 (696.7) |  | 49 | 63-960 | 433.0 (218.1) |
| A2 | 38 | 54-1621 | 581.4 (456.5) |  | 30 | 50-507 | 234.4 (141.0) |  | 47 | 53-1805 | 483.4 (456.4) |  | 32 | 54-625 | 217.8 (149.5) |
| A3 | 27 | 51-964 | 247.6 (207.9) |  | 15 | 57-331 | 123.1 (69.0) |  | 28 | 52-1465 | 225.4 (273.6) |  | 13 | 50-506 | 122.5 (121.0) |
| A | 43 | 170-3180 | 1796.2 (708.5) |  | 42 | 125-1037 | 607.8 (222.6) |  | 51 | 232-3040 | 1856.3 (782.2) |  | 49 | 100-1006 | 628.9 (238.4) |
| B1 | 42 | 84-2418 | 741.3 (563.1) |  | 38 | 60-805 | 271.7 (185.0) |  | 49 | 61-2229 | 717.0 (566.1) |  | 43 | 61-733 | 278.8 (183.7) |
| B2/3 | 39 | 77-1146 | 494.2 (308.0) |  | 34 | 50-401 | 176.0 (97.5) |  | 45 | 60-2050 | 525.8 (522.2) |  | 36 | 52-688 | 216.3 (183.3) |
| C | 22 | 55-913 | 239.7 (209.9) |  | 12 | 50-299 | 103.8 (70.5) |  | 19 | 62-719 | 169.7 (154.7) |  | 7 | 53-235 | 93.3 (64.9) |
| B2/3&C | 39 | 127-1630 | 637.4 (404.0) |  | 35 | 57-524 | 216.2 (124.6) |  | 45 | 60-2127 | 606.0 (554.5) |  | 36 | 52-712 | 244.6 (190.0) |

n shows the number of subjects reaching the criterion of at least 50 epochs in the respective EEG-vigilance stage. Additionally, corresponding mean and range of remaining epochs are given. Standard deviations are shown in the parentheses.

Table S2. Number of included subjects for EEG-vigilance stage comparisons

|  | **pair** | **ignored condition** | |  | **attended condition** | |
| --- | --- | --- | --- | --- | --- | --- |
|  |  | **standard stimuli** | **deviant stimuli** |  | **standard stimuli** | **deviant stimuli** |
| repeated measure ANOVA | A vs. B1 vs. B2/3&C | 38 | 31 |  | 44 | 29 |
| paired t-tests | 0 vs. A1 | 22 | 17 |  | 31 | 21 |
|  | 0 vs. A2 | 19 | 10 |  | 27 | 11 |
|  | 0 vs. A3 | 13 | (5) |  | 13 | (3) |
|  | A1 vs. A2 | 35 | 28 |  | 47 | 32 |
|  | A1 vs. A3 | 24 | 12 |  | 28 | 12 |
|  | A2 vs. A3 | 26 | 13 |  | 28 | 12 |
|  | B1 vs. B2/3 | 38 | 31 |  | 44 | 30 |
|  | B1 vs. C | 22 | 12 |  | 19 | (7) |
|  | B2/3 vs. C | 22 | 11 |  | 19 | (7) |

Note that each comparison is based on the same subjects. Some comparisons have low number of subjects (comparisons below ten are put in parentheses and were not analyzed)
