# Supplementary material for: Evoked potentials and behavioral performance during different states of brain arousal
Source: BMC Neurosci. 2017 Jan 25;18:21. doi: 10.1186/s12868-017-0340-9 (PMC5267455; doi:10.1186/s12868-017-0340-9)
Supplement: Supplementary file 3 — Additional file 3. Grand average waveforms for deviant stimuli and results of multiple comparisons. [file 12868_2017_340_MOESM3_ESM.docx]

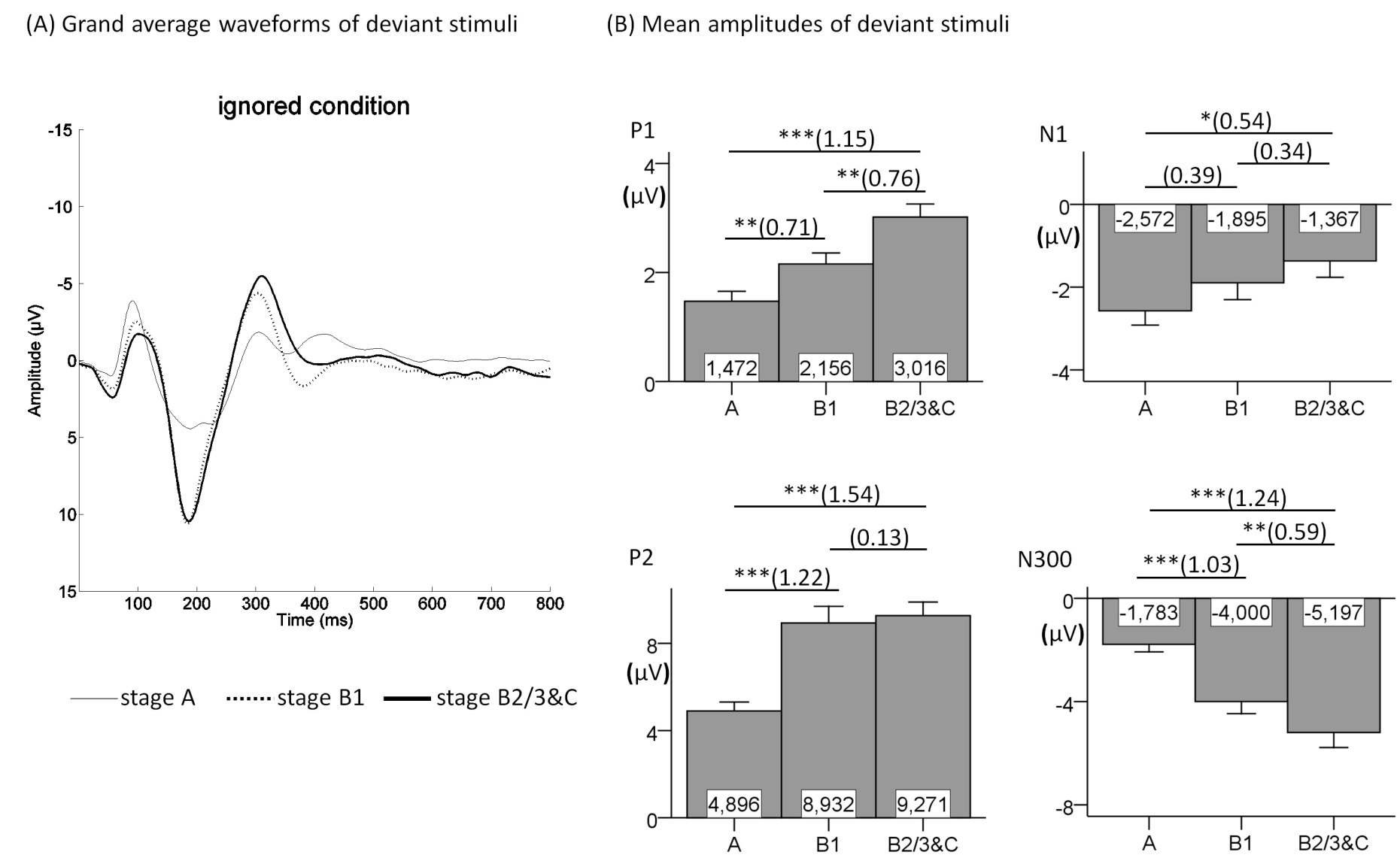


**Figure S1.** **Grand average waveforms (A) and mean amplitudes (B) for deviant components in the ignored condition.**

The deviant P1, N1, P2 and N300 are presented at Cz electrode in EEG-vigilance stages A, B1 and B2/3&C (N = 33). The main effects of EEG-vigilance stages on the deviant P1 [*F*_(2,60)_ = 27.632, *p* < .001, η_p_² = 0.479], N1 [*F*_(1.666,49.992)_ = 6.457, *p* < .01, η_p_² = 0.177], P2 [*F*_(2,60)_ = 42.459, *p* < .001, η_p_² = 0.586] and N300 [*F*_(2,60)_ = 34.122, *p* < .001, η_p_² = 0.532] were significant. The significant results of multiple comparisons are marked with asterisk (* *p* < .05; ** *p* < .01; *** *p* < .001; each p-value is Bonferroni corrected). The corresponding effect sizes for Cohen’s *dz* are presented in parentheses. Neither grand average waveform nor mean amplitude for the MMN [*F*_(2,60)_ = .126, *p* = .882, η_p_² = 0.004] is represented since no effect was found.


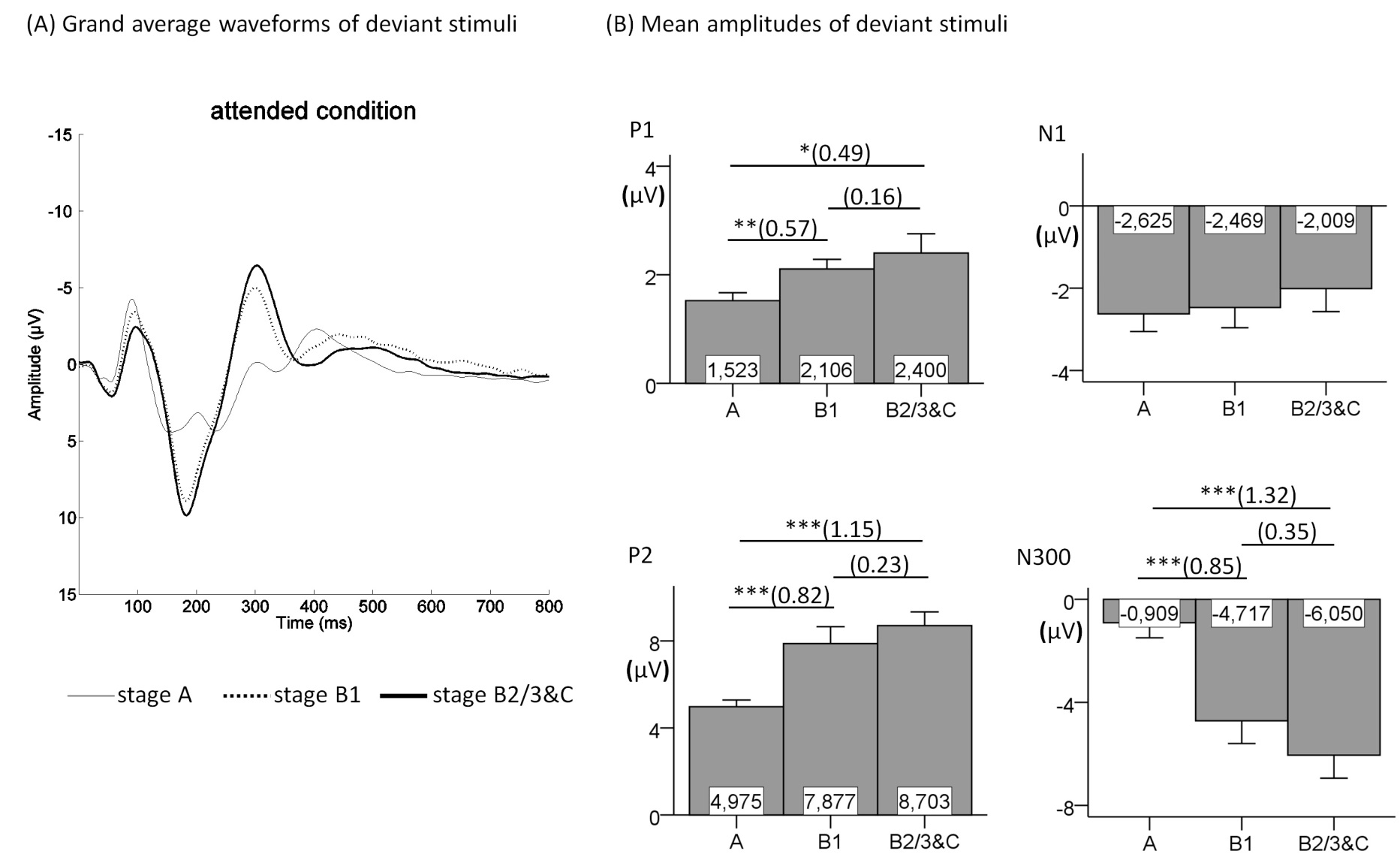


**Figure S2.** **Grand average waveforms (A) and mean amplitudes (B) for deviant components in the attended condition.**

The deviant P1, N1, P2 and N300 are presented at Cz electrode in EEG-vigilance stages A, B1 and B2/3&C (N = 29). Deviant P1 [*F*_(1.475,41.291)_ = 4.483, *p* < .05, η_p_² = 0.138], P2 [*F*_(2,56)_ = 21.613, *p* < .001, η_p_² = 0.436] and N300 [*F*_(2,56)_ = 24.853, *p* < .001, η_p_² = 0.470] differed significantly between EEG-vigilance stages. The significant results of multiple comparisons are marked with asterisk (* *p* < .05; ** *p* < .01; *** *p* < .001; each p-value is Bonferroni corrected). The corresponding effect sizes for Cohen’s *dz* are presented in parentheses. No multiple comparisons were run for deviant N1 [*F*_(2,56)_ = 2.102, *p* = .132, η_p_² = 0.070] since no effect was found. Neither grand average waveform nor mean amplitude for the P3 [*F*_(2,56)_ = 1.416, *p* = .251, η_p_² = 0.048] is represented since no effect was found.
