# Supplementary material for: Evoked potentials and behavioral performance during different states of brain arousal
Source: BMC Neurosci. 2017 Jan 25;18:21. doi: 10.1186/s12868-017-0340-9 (PMC5267455; doi:10.1186/s12868-017-0340-9)
Supplement: Supplementary file 4 — Additional file 4. Paired sample t-tests for comparisons of EPs and behavioral performance between EEG-vigilance (sub-)stages. [file 12868_2017_340_MOESM4_ESM.docx]

Table S3. Results of paired sample t-tests for EPs and behavioral performance between 0 and A-substages

|  | **pair** | **ignored condition** | | | | |  | **attended condition** | | | | |
| --- | --- | --- | --- | --- | --- | --- | --- | --- | --- | --- | --- | --- |
|  |  | **mean** | ***df*** | ***t*** | ***p*** | ***dz*** |  | **mean** | ***df*** | ***t*** | ***p*** | ***dz*** |
| standard P1 | 0 vs. A1 | 1.9 (1.2) vs. 1.7 (1.1) | 21 | 1.503 | .148 | 0.32 |  | 1.7 (0.9) vs. 1.5 (0.8) | 30 | 1.144 | .262 | 0.21 |
|  | 0 vs. A2 | 1.7 (1.2) vs. 1.6 (1.2) | 18 | 0.639 | .531 | 0.15 |  | 1.7 (0.9) vs. 1.4 (0.9) | 26 | 1.307 | .203 | 0.25 |
|  | 0 vs. A3 | 1.8 (1.2) vs. 2.3 (1.7) | 12 | -1.022 | .327 | 0.28 |  | 1.6 (0.9) vs. 2.0 (1.0) | 12 | -1.631 | .129 | 0.45 |
| standard N1 | 0 vs. A1 | -0.9 (1.7) vs. -0.7 (1.3) | 21 | -1.130 | .271 | 0.24 |  | -0.9 (1.7) vs. -1.0 (1.5) | 30 | 0.957 | .346 | 0.17 |
|  | 0 vs. A2 | -0.8 (1.3) vs. -0.8 (1.6) | 18 | -0.263 | .795 | 0.06 |  | -0.9 (1.7) vs. -1.1 (1.6) | 26 | 1.010 | .322 | 0.19 |
|  | 0 vs. A3 | -0.8 (1.5) vs. -0.6 (1.8) | 12 | -0.540 | .599 | 0.15 |  | -1.1 (1.6) vs. -1.3 (2.1) | 12 | 0.600 | .560 | 0.17 |
| standard P2 | 0 vs. A1 | 4.5 (2.2) vs. 4.3 (1.8) | 21 | 0.876 | .391 | 0.19 |  | 3.8 (1.3) vs. 4.2 (1.3) | 30 | -3.155^**^ | .004 | 0.57 |
|  | 0 vs. A2 | 4.6 (2.0) vs. 4.8 (2.0) | 18 | -0.500 | .623 | 0.11 |  | 4.0 (1.3) vs. 4.5 (1.3) | 26 | -2.435^*^ | .022 | 0.47 |
|  | 0 vs. A3 | 4.6 (1.6) vs. 4.7 (1.7) | 12 | -0.227 | .824 | 0.06 |  | 3.9 (1.2) vs. 4.7 (1.9) | 12 | -2.724^*^ | .018 | 0.75 |
| standard N300 | 0 vs. A1 | -2.1 (1.7) vs. -1.9 (1.5) | 21 | -0.911 | .373 | 0.19 |  | -1.8 (2.0) vs. -1.8 (1.6) | 30 | -0.462 | .647 | 0.08 |
|  | 0 vs. A2 | -1.9 (1.6) vs. -2.0 (1.4) | 18 | 0.205 | .840 | 0.05 |  | -1.5 (1.9) vs. -1.8 (2.0) | 26 | 1.300 | .205 | 0.25 |
|  | 0 vs. A3 | -1.8 (1.8) vs. -1.7 (2.0) | 12 | -0.554 | .590 | 0.15 |  | -1.5 (2.3) vs. -2.2 (2.5) | 12 | 1.987 | .070 | 0.55 |
| deviant P1 | 0 vs. A1 | 2.1 (1.5) vs. 1.7 (1.0) | 16 | 1.346 | .197 | 0.33 |  | 1.7 (1.4) vs. 1.4 (0.8) | 20 | 1.036 | .312 | 0.23 |
|  | 0 vs. A2 | 1.9(1.5) vs. 1.3 (1.5) | 9 | 1.539 | .158 | 0.49 |  | 1.9 (1.4) vs. 1.9 (1.4) | 10 | 0.018 | .986 | 0.01 |
| deviant N1 | 0 vs. A1 | -2.3 (1.8) vs. -2.1 (1.8) | 16 | -1.224 | .239 | 0.30 |  | -2.5 (2.6) vs. -2.5 (2.5) | 20 | -0.009 | .993 | 0.01 |
|  | 0 vs. A2 | -3.2 (1.7) vs. -3.2 (1.7) | 9 | 0.222 | .829 | 0.07 |  | -2.6 (2.3) vs. -2.6 (2.1) | 10 | 0.103 | .920 | 0.03 |
| deviant P2 | 0 vs. A1 | 4.0 (1.7) vs. 4.4 (1.7) | 16 | -1.456 | .165 | 0.35 |  | 4.4 (2.9) vs. 4.5 (2.3) | 20 | -0.248 | .807 | 0.05 |
|  | 0 vs. A2 | 4.5 (2.0) vs. 4.3 (2.1) | 9 | 0.225 | .827 | 0.07 |  | 4.6 (3.7) vs. 4.2 (2.7) | 10 | 0.933 | .373 | 0.28 |
| deviant N300 | 0 vs. A1 | -2.5 (1.4) vs. -2.0 (1.3) | 16 | -1.627 | .123 | 0.39 |  | -0.6 (4.1) vs. -0.6 (3.1) | 20 | 0.054 | .957 | 0.01 |
|  | 0 vs. A2 | -2.1 (1.3) vs. -2.1 (2.6) | 9 | -0.133 | .897 | 0.04 |  | 0.6 (5.0) vs. 0.4 (3.5) | 10 | 0.181 | .860 | 0.05 |
| RT (ms) | 0 vs. A1 |  |  |  |  |  |  | 467.6 (84.2) vs. 472.0 (78.7) | 20 | -0.563 | .580 | 0.12 |
|  | 0 vs. A2 |  |  |  |  |  |  | 426.9 (68.5) vs. 426.2 (74.8) | 10 | 0.103 | .920 | 0.03 |
| HR (%) | 0 vs. A1 |  |  |  |  |  |  | 83.2 (21.1) vs. 84.8 (18.0) | 20 | -0.794 | .437 | 0.17 |
|  | 0 vs. A2 |  |  |  |  |  |  | 91.6 (8.4) vs. 91.9 (9.9) | 10 | -0.171 | .867 | 0.05 |
| OR (%) | 0 vs. A1 |  |  |  |  |  |  | 14.4 (20.2) vs. 13.6 (18.1) | 20 | 0.488 | .631 | 0.11 |
|  | 0 vs. A2 |  |  |  |  |  |  | 6.6 (7.6) vs. 6.4 (8.9) | 10 | 0.153 | .882 | 0.05 |
| FAR (%) | 0 vs. A1 |  |  |  |  |  |  | 0.7 (1.6) vs. 0.3 (0.4) | 20 | 1.244 | .228 | 0.27 |
|  | 0 vs. A2 |  |  |  |  |  |  | 0.3 (0.2) vs. 0.3 (0.3) | 10 | 1.411 | .189 | 0.43 |

Note that comparison between 0 and A3 for deviant stimuli was not calculated since number of subjects was insufficient (N < 10).

Standard deviations are shown in the parentheses.

* *p* < .05

** *p* < .01

Table S4. Results of paired sample t-tests for EPs and behavioral performance between A-substages

|  | **pair** | **ignored condition** | | | | |  |  | **attended condition** | | | |  |
| --- | --- | --- | --- | --- | --- | --- | --- | --- | --- | --- | --- | --- | --- |
|  |  | **mean** | ***df*** | ***t*** | | ***p*** | ***dz*** |  | **mean** | ***df*** | ***t*** | ***p*** | ***dz*** |
| standard P1 | A1 vs. A2 | 1.3 (1.1) vs. 1.4 (1.0) | 34 | -0.745 | | .461 | 0.13 |  | 1.5 (0.8) vs. 1.4 (1.0) | 46 | 0.103 | .918 | 0.01 |
|  | A1 vs. A3 | 1.3 (1.1) vs. 2.0 (1.5) | 23 | -2.430^*^ | | .023 | 0.50 |  | 1.5 (0.9) vs. 2.1 (1.0) | 27 | -3.029^**^ | .005 | 0.57 |
|  | A2 vs. A3 | 1.4 (1.0) vs. 1.8 (1.4) | 25 | -1.439 | | .163 | 0.28 |  | 1.4 (1.0) vs. 2.1 (1.0) | 27 | -3.333^**^ | .002 | 0.63 |
| standard N1 | A1 vs. A2 | -1.1 (1.6) vs. -1.1 (1.8) | 34 | 0.417 | | .680 | 0.07 |  | -1.2 (1.9) vs. -1.2 (1.9) | 46 | -0.670 | .506 | 0.10 |
|  | A1 vs. A3 | -1.0 (1.6) vs. -0.8 (1.8) | 23 | -0.970 | | .342 | 0.20 |  | -1.4 (2.0) vs. -0.9 (2.1) | 27 | -2.065^*^ | .049 | 0.39 |
|  | A2 vs. A3 | -1.1 (1.9) vs. -0.9 (1.7) | 25 | -0.921 | | .366 | 0.18 |  | -1.2 (1.9) vs. -0.9 (2.1) | 27 | -1.133 | .267 | 0.21 |
| standard P2 | A1 vs. A2 | 3.8 (1.7) vs. 4.1 (2.0) | 34 | -1.294 | | .204 | 0.22 |  | 4.4 (1.5) vs. 4.8 (1.8) | 46 | -2.235^*^ | .030 | 0.33 |
|  | A1 vs. A3 | 3.9 (1.8) vs. 4.4 (1.4) | 23 | -2.457^*^ | | .022 | 0.50 |  | 4.5 (1.5) vs. 5.1 (1.9) | 27 | -2.522^*^ | .018 | 0.48 |
|  | A2 vs. A3 | 4.0 (1.7) vs. 4.3 (1.4) | 25 | -1.442 | | .162 | 0.28 |  | 4.8 (1.5) vs. 5.1 (1.9) | 27 | -1.499 | .146 | 0.28 |
| standard N300 | A1 vs. A2 | -1.9 (1.3) vs. -2.2 (1.4) | 34 | 2.608^*^ | | .013 | 0.44 |  | -1.7 (1.2) vs. -1.9 (1.7) | 46 | 1.207 | .234 | 0.18 |
|  | A1 vs. A3 | -2.0 (1.5) vs. -1.8 (2.0) | 23 | -0.360 | | .722 | 0.07 |  | -1.7 (1.3) vs. -2.1 (2.3) | 27 | 1.388 | .176 | 0.26 |
|  | A2 vs. A3 | -2.2 (1.5) vs. -2.1 (1.7) | 25 | -0.645 | | .525 | 0.13 |  | -1.8 (1.7) vs. -2.1 (2.3) | 27 | 1.342 | .191 | 0.25 |
| deviant P1 | A1 vs. A2 | 1.1 (1.2) vs. 1.5 (1.3) | 27 | -1.919 | | .066 | 0.36 |  | 1.6 (1.1) vs. 1.8 (1.3) | 31 | -0.890 | .380 | 0.16 |
|  | A1 vs. A3 | 1.1 (1.5) vs. 1.9 (1.6) | 11 | -1.643 | | .129 | 0.47 |  | 2.0 (1.4) vs. 1.5 (1.6) | 12 | 1.177 | .262 | 0.33 |
|  | A2 vs. A3 | 1.4 (1.4) vs. 1.9 (1.7) | 12 | -1.221 | | .245 | 0.34 |  | 1.8 (1.3) vs. 1.5 (1.6) | 12 | 0.829 | .423 | 0.23 |
| deviant N1 | A1 vs. A2 | -2.9 (2.0) vs. -3.3 (2.1) | 27 | 1.838 | | .077 | 0.35 |  | -2.8 (2.1) vs. -2.8 (2.3) | 31 | 0.490 | .628 | 0.09 |
|  | A1 vs. A3 | -2.2 (2.0) vs. -1.7 (2.1) | 11 | -1.991 | | .072 | 0.57 |  | -3.0 (2.4) vs. -2.7 (2.1) | 12 | -0.818 | .429 | 0.23 |
|  | A2 vs. A3 | -3.1 (2.0) vs. -2.4 (1.9) | 12 | -2.694^*^ | | .020 | 0.75 |  | -3.1 (2.8) vs. -2.7 (2.1) | 12 | -1.015 | .330 | 0.28 |
| deviant P2 | A1 vs. A2 | 4.6 (2.1) vs. 4.9 (2.2) | 27 | -1.193 | | .243 | 0.23 |  | 4.1 (2.4) vs. 4.5 (2.4) | 31 | -1.981 | .057 | 0.35 |
|  | A1 vs. A3 | 4.9 (1.4) vs. 5.4 (1.8) | 11 | -1.179 | | .263 | 0.34 |  | 4.6 (3.2) vs. 5.3 (3.2) | 12 | -1.848 | .089 | 0.51 |
|  | A2 vs. A3 | 4.9 (2.1) vs. 5.3 (1.9) | 12 | -0.814 | | .432 | 0.23 |  | 5.2 (2.8) vs. 5.3 (3.2) | 12 | -0.401 | .696 | 0.11 |
| deviant N300 | A1 vs. A2 | -1.5 (1.6) vs. -1.9 (2.1) | 27 | 1.369 | | .182 | 0.26 |  | -0.8 (3.6) vs. -1.4 (3.4) | 31 | 2.283^*^ | .029 | 0.40 |
|  | A1 vs. A3 | -1.2 (1.4) vs. -1.0 (1.5) | 11 | -0.676 | | .513 | 0.20 |  | -1.3 (2.9) vs. -2.2 (4.2) | 12 | 1.481 | .164 | 0.41 |
|  | A2 vs. A3 | -1.4 (1.4) vs. -1.3 (1.7) | 12 | -0.179 | | .861 | 0.05 |  | -1.8 (3.3) vs. -2.2 (4.2) | 12 | 0.780 | .451 | 0.22 |
| RT (ms) | A1 vs. A2 |  |  |  | |  |  |  | 458.5 (73.4) vs. 458.0 (74.9) | 31 | 0.166 | .869 | 0.03 |
|  | A1 vs. A3 |  |  |  | |  |  |  | 469.2 (59.9) vs. 479.3 (78.6) | 12 | -1.071 | .305 | 0.30 |
|  | A2 vs. A3 |  |  |  |  |  |  |  | 469.6 (69.0) vs. 479.3 (78.6) | 12 | -1.242 | .238 | 0.35 |
| HR (%) | A1 vs. A2 |  |  |  |  |  |  |  | 91.1 (11.9) vs. 90.2 (12.2) | 31 | 2.116^*^ | .042 | 0.37 |
|  | A1 vs. A3 |  |  |  |  |  |  |  | 91.4 (9.2) vs. 87.2 (13.0) | 12 | 2.853^*^ | .015 | 0.79 |
|  | A2 vs. A3 |  |  |  |  |  |  |  | 90.0 (10.2) vs. 87.2 (13.0) | 12 | 1.885 | .084 | 0.52 |
| OR (%) | A1 vs. A2 |  |  |  |  |  |  |  | 7.4 (10.3) vs. 7.7 (10.4) | 31 | -0.756 | .455 | 0.13 |
|  | A1 vs. A3 |  |  |  |  |  |  |  | 7.5 (8.3) vs. 10.4 (12.3) | 12 | -1.962 | .073 | 0.54 |
|  | A2 vs. A3 |  |  |  |  |  |  |  | 8.6 (9.2) vs. 10.4 (12.3) | 12 | -1.189 | .257 | 0.33 |
| FAR (%) | A1 vs. A2 |  |  |  |  |  |  |  | 0.4 (0.4) vs. 0.4 (0.6) | 31 | -1.321 | .196 | 0.24 |
|  | A1 vs. A3 |  |  |  |  |  |  |  | 0.4 (0.4) vs. 0.5 (0.5) | 12 | -0.772 | .455 | 0.96 |
|  | A2 vs. A3 |  |  |  |  |  |  |  | 0.4 (0.3) vs. 0.5 (0.5) | 12 | -1.242 | .238 | 0.34 |

Standard deviations are shown in the parentheses.

* *p* < .05

** *p* < .01

Table S5. Results of paired sample t-tests for EPs and behavioral performance between B1, B2/3 and C

|  | **pair** | **ignored condition** | | | | |  |  | **attended condition** | | | |  |
| --- | --- | --- | --- | --- | --- | --- | --- | --- | --- | --- | --- | --- | --- |
|  |  | **mean** | ***df*** | ***t*** | | ***p*** | ***dz*** |  | **mean** | ***df*** | ***t*** | ***p*** | ***dz*** |
| standard P1 | B1 vs. B2/3 | 2.0 (1.0) vs. 2.4 (1.2) | 37 | -2.226^*^ | | .032 | 0.36 |  | 2.3 (1.1) vs. 2.5 (1.4) | 43 | -0.887 | .380 | 0.13 |
|  | B1 vs. C | 2.0 (0.9) vs. 3.1 (1.6) | 21 | -3.118^**^ | | .005 | 0.69 |  | 2.1 (0.9) vs. 3.4 (1.5) | 18 | -3.431^**^ | .003 | 0.79 |
|  | B2/3 vs. C | 2.7 (1.2) vs. 3.1 (1.6) | 21 | -1.040 | | .310 | 0.22 |  | 2.9 (1.6) vs. 3.4 (1.5) | 18 | -1.138 | .270 | 0.26 |
| standard N1 | B1 vs. B2/3 | -0.4 (1.8) vs. 0.3 (1.9) | 37 | -4.065^***^ | | .000 | 0.66 |  | -0.5 (1.8) vs. 0.0 (2.0) | 43 | -3.166^**^ | .003 | 0.48 |
|  | B1 vs. C | -0.1 (1.6) vs. 1.3 (2.0) | 21 | -4.999^***^ | | .000 | 1.07 |  | -0.3 (1.9) vs. 0.6 (1.8) | 18 | -3.022^**^ | .007 | 0.69 |
|  | B2/3 vs. C | 0.7 (1.7) vs. 1.3 (2.0) | 21 | -1.979 | | .061 | 0.42 |  | 0.4 (1.8) vs. 0.6 (1.8) | 18 | -0.404 | .691 | 0.10 |
| standard P2 | B1 vs. B2/3 | 5.8 (2.9) vs. 6.1 (3.0) | 37 | -0.640 | | .526 | 0.10 |  | 5.7 (3.0) vs. 6.2 (3.0) | 43 | -1.126 | .267 | 0.17 |
|  | B1 vs. C | 6.3 (2.7) vs. 6.5 (3.0) | 21 | -0.385 | | .704 | 0.08 |  | 5.3 (3.5) vs. 5.6 (3.1) | 18 | -0.502 | .622 | 0.12 |
|  | B2/3 vs. C | 6.6 (3.0) vs. 6.5 (3.0) | 21 | 0.151 | | .882 | 0.03 |  | 5.9 (3.4) vs. 5.6 (3.1) | 18 | 0.635 | .533 | 0.15 |
| standard N300 | B1 vs. B2/3 | -4.0 (2.1) vs. -4.5 (2.4) | 37 | 2.144^*^ | | .039 | 0.35 |  | -4.0 (2.4) vs. -4.7 (2.8) | 43 | 2.416^*^ | .020 | 0.36 |
|  | B1 vs. C | -4.4 (2.2) vs. -6.4 (4.1) | 21 | 3.032^**^ | | .006 | 0.65 |  | -4.4 (2.1) vs. -6.0 (3.0) | 18 | 2.754^*^ | .013 | 0.63 |
|  | B2/3 vs. C | -5.2 (2.7) vs. -6.4 (4.1) | 21 | 2.062 | | .052 | 0.44 |  | -5.4 (2.7) vs. -6.0 (3.0) | 19 | 0.938 | .361 | 0.21 |
| deviant P1 | B1 vs. B2/3 | 2.1 (1.1) vs. 2.8 (1.4) | 30 | -2.801^**^ | | .009 | 0.50 |  | 2.1 (1.0) vs. 2.5 (1.9) | 29 | -1.273 | .213 | 0.23 |
|  | B1 vs. C | 2.0 (0.8) vs. 3.6 (1.5) | 11 | -5.013^***^ | | .000 | 1.45 |  |  |  |  |  |  |
|  | B2/3 vs. C | 2.4 (1.2) vs. 3.6 (1.5) | 10 | -2.180 | | .054 | 0.66 |  |  |  |  |  |  |
| deviant N1 | B1 vs. B2/3 | -1.9 (2.3) vs. -1.7 (2.2) | 30 | -0.771 | | .447 | 0.14 |  | -2.5 (2.7) vs. -1.8 (3.0) | 29 | -2.221^*^ | .034 | 0.41 |
|  | B1 vs. C | -2.0 (2.7) vs. 0.3 (1.7) | 11 | -3.328^**^ | | .007 | 0.96 |  |  |  |  |  |  |
|  | B2/3 vs. C | -1.6 (1.8) vs. 0.2 (1.8) | 10 | -2.493^*^ | | .032 | 0.75 |  |  |  |  |  |  |
| deviant P2 | B1 vs. B2/3 | 9.1 (4.1) vs. 9.3 (3.4) | 30 | -0.511 | | .613 | 0.08 |  | 8.0 (4.1) vs. 9.0 (3.5) | 29 | -1.688 | .102 | 0.31 |
|  | B1 vs. C | 8.6 (2.9) vs. 10.1 (3.2) | 11 | -1.423 | | .182 | 0.41 |  |  |  |  |  |  |
|  | B2/3 vs. C | 10.2 (1.9) vs. 10.2 (3.3) | 10 | -0.034 | | .974 | 0.01 |  |  |  |  |  |  |
| deviant N300 | B1 vs. B2/3 | -3.8 (2.7) vs. -5.2 (3.4) | 30 | 3.855^**^ | | .001 | 0.69 |  | -4.7 (4.7) vs. -5.9 (4.7) | 29 | 1.799 | .082 | 0.33 |
|  | B1 vs. C | -4.7 (3.2) vs. -6.1 (3.7) | 11 | 1.384 | | .194 | 0.40 |  |  |  |  |  |  |
|  | B2/3 vs. C | -7.2 (3.4) vs. -5.9 (3.8) | 10 | -1.461 | | .175 | 0.44 |  |  |  |  |  |  |
| RT (ms) | B1 vs. B2/3 |  |  |  |  |  |  |  | 557.4 (93.3) vs. 567.9 (84.2) | 29 | -0.864 | .395 | 0.16 |
| HR (%) | B1 vs. B2/3 |  |  |  |  |  |  |  | 55.3 (24.9) vs. 39.4 (24.7) | 29 | 3.344^**^ | .002 | 0.61 |
| OR (%) | B1 vs. B2/3 |  |  |  |  |  |  |  | 39.9 (23.7) vs. 56.4 (24.9) | 29 | -3.704^**^ | .001 | 0.68 |
| FAR (%) | B1 vs. B2/3 |  |  |  |  |  |  |  | 1.9 (2.0) vs. 1.2 (1.2) | 29 | 2.432^*^ | .021 | 0.44 |

Note that comparisons with stage C for deviant stimuli in the attended condition were not calculated since number of subjects was insufficient (N < 10).

Standard deviations are shown in the parentheses.

* *p* < .05

** *p* < .01

*** *p* < .001

Table S6. Results of paired sample t-tests for EPs and behavioral performance between 0 and B1

|  | **ignored condition** | | | | |  | **attended condition** | | | | |
| --- | --- | --- | --- | --- | --- | --- | --- | --- | --- | --- | --- |
|  | **mean** | ***df*** | ***t*** | ***p*** | ***dz*** |  | **mean** | ***df*** | ***t*** | ***p*** | ***dz*** |
| standard P1 | 1.9 (1.2) vs. 2.0 (1.2) | 23 | -0.504 | .619 | 0.10 |  | 1.7 (0.8) vs. 2.1 (1.0) | 29 | -2.881^**^ | .007 | 0.53 |
| standard N1 | -0.9 (1.7)vs. -0.3 (1.3) | 23 | -3.002^**^ | .006 | 0.61 |  | -.9 (1.7) vs. -0.6 (1.6) | 29 | -1.142 | .263 | 0.21 |
| standard P2 | 4.6 (2.1) vs. 5.7 (2.9) | 23 | -2.888^**^ | .008 | 0.59 |  | 3.9 (1.3) vs. 4.8 (2.2) | 29 | -3.307^**^ | .003 | 0.60 |
| standard N300 | -2.1 (1.6) vs. -3.3 (1.8) | 23 | 3.586^**^ | .002 | 0.73 |  | -1.8 (2.1) vs. -3.0 (1.9) | 29 | 5.600^***^ | .000 | 1.02 |
| deviant P1 | 1.8 (1.4) vs. 2.1 (1.1) | 16 | -1.690 | .110 | 0.41 |  | 1.7 (1.4) vs. 2.0 (1.0) | 20 | -1.244 | .228 | 0.27 |
| deviant N1 | -2.1 (1.3) vs. -1.3 (1.3) | 16 | -2.040 | .058 | 0.49 |  | -2.7 (2.5) vs. -3.0 (2.7) | 20 | 1.271 | .218 | 0.28 |
| deviant P2 | 4.2 (1.8) vs. 6.6 (3.4) | 16 | -3.711^**^ | .002 | 0.90 |  | 4.4 (2.9) vs. 5.2 (3.6) | 20 | -2.315^*^ | .031 | 0.50 |
| deviant N300 | -2.6 (1.4) vs. -3.7 (2.0) | 16 | 2.273^*^ | .037 | 0.55 |  | -0.4 (3.9) vs. -2.5 (3.2) | 20 | 4.909^***^ | .000 | 1.07 |
| RT (ms) |  |  |  |  |  |  | 465.5 (86.0) vs. 519.7 (98.4) | 20 | -5.838^***^ | .000 | 1.27 |
| HR (%) |  |  |  |  |  |  | 83.9 (21.4) vs. 68.9 (24.4) | 20 | 3.940^**^ | .001 | 0.86 |
| OR (%) |  |  |  |  |  |  | 13.9 (20.5) vs. 27.1 (23.9) | 20 | -3.579^**^ | .002 | 0.78 |
| FAR (%) |  |  |  |  |  |  | 0.7 (1.6) vs. 1.5 (2.3) | 20 | -1.986 | .061 | 0.43 |

Standard deviations are shown in the parentheses.

* *p* < .05

** *p* < .01

*** *p* < .001
