# Supplementary material for: Evoked potentials and behavioral performance during different states of brain arousal
Source: BMC Neurosci. 2017 Jan 25;18:21. doi: 10.1186/s12868-017-0340-9 (PMC5267455; doi:10.1186/s12868-017-0340-9)
Supplement: Supplementary file 5 — Additional file 5. Mean number of epochs in EEG-vigilance stages during 4 time blocks. [file 12868_2017_340_MOESM5_ESM.docx]

Table S7. Mean number of epochs in EEG-vigilance stages during 4 time blocks in ignored and attended conditions

| **EEG-vigilance stage** | **ignored condition** | | | |  | **attended condition** | | | |
| --- | --- | --- | --- | --- | --- | --- | --- | --- | --- |
|  | **Block 1** | **Block 2** | **Block3** | **Block 4** |  | **Block 1** | **Block 2** | **Block3** | **Block 4** |
| 0 | 66.9 (79.4) | 44.8 (59.9) | 53.7 (61.0) | 55.5 (75.0) |  | 76.2 (108.1) | 51.7 (65.7) | 74.0 (125.2) | 92.4 (125.5) |
| A1 | 280.8 (183.9) | 236.4 (176.3) | 273.3 (189.1) | 313.9 (181.5) |  | 334.1 (191.6) | 255.7 (162.3) | 318.3 (204.8) | 345.9 (193.6) |
| A2 | 135.9 (121.8) | 111.7 (109.0) | 124.0 (116.2) | 134.4 (124.1) |  | 117.0 (128.1) | 111.0 (126.7) | 111.7 (116.5) | 105.5 (106.9) |
| A3 | 48.8 (51.9) | 43.4 (49.4) | 46.8 (50.2) | 44.3 (53.1) |  | 37.0 (58.9) | 39.1 (59.3) | 34.7 (51.2) | 37.9 (66.4) |
| B1 | 194.1 (159.9) | 193.8 (149.0) | 183.7 (132.7) | 163.4 (150.5) |  | 197.2 (153.4) | 204.5 (166.9) | 154.0 (153.3) | 160.8 (142.0) |
| B2/3 | 97.4 (85.7) | 152.8 (100.4) | 112.7 (105.4) | 96.1 (97.3) |  | 98.2 (110.7) | 159.1 (162.8) | 130.2 (151.2) | 99.8 (120.0) |
| C | 66.3 (63.1) | 65.5 (61.3) | 99.6 (85.6) | 60.7 (56.0) |  | 42.1 (23.8) | 82.0 (55.0) | 52.8 (53.5) | 68.1 (62.0) |

Standard deviations are shown in the parentheses.
